# The efficacy of cryolipolysis treatment on arms and inner thighs

**DOI:** 10.1007/s10103-015-1781-y

**Published:** 2015-06-23

**Authors:** Rungsima Wanitphakdeedecha, Angkana Sathaworawong, Woraphong Manuskiatti

**Affiliations:** Department of Dermatology, Faculty of Medicine Siriraj Hospital, Mahidol University, 2 Pran-nok Road, Bangkok, 10700 Thailand

**Keywords:** Cryolipolysis, Arms, Inner thighs

## Abstract

Cryolipolysis has emerged as a new non-invasive body contouring method using controlled cooling to selectively destroy fat cells. Previous studies demonstrated the safety and efficacy of cryolipolysis for the reduction of localized subcutaneous fat on abdomen and flanks. Recently, the new flat cup vacuum applicator has been developed to treat localized subcutaneous fat on arms and inner thighs. The objective of this study was to determine the efficacy of non-invasive cryolipolysis for body contouring with a flat cup vacuum applicator on arms and inner thighs. Twenty females with excess localized subcutaneous fat on arms or inner thighs received a single cryolipolysis treatment. Forty treatment areas have been treated including 10 arms and 30 inner thighs. Subjects were evaluated using standardized photographs and measurements of body weight and circumference of arms or inner thighs at baseline, 3-month, and 6-month follow-up visits. Physicians’ evaluation and patient’s satisfaction of clinical improvement were also measured. Of all 20 subjects, 17 (10 arms and 24 inner thighs) completed the treatment protocol and attended all follow-up visits. Three subjects were withdrawn from the study, 1 subject could not complete the treatment session due to pain and numbness during treatment, 1 subject became pregnant after treatment, and the other subject could not attend all required follow-up visits. There was significant circumference reduction of 0.41 and 0.72 cm at 3-month and 6-month follow-up visits (*p* = 0.017), respectively. Most of the patients were rated to have 1–25 % improvement at 6 months after treatment and were satisfied with the treatment outcome. The new cryolipolysis flat cup vacuum applicator provided beneficial effects for circumferential reduction of arms and inner thighs.

## Introduction

With the rising demand for fat reduction, multiple non-invasive technologies have grown dramatically over the past decade among patients who want to avoid the surgical risks from liposuction [1]. Multiple treatment modalities, including bipolar radiofrequency with infrared heat and pulsatile suction, multipolar radiofrequency, laser, and ultrasound devices, have been employed with variable results [2, 3]. Only some of these procedures have been approved by the US Food and Drug Administration for selective fat reduction. Clinical studies of low-level laser therapy [4], high-intensity focused ultrasound [5], and cryolipolysis [6] revealed the safety and efficacy of these technologies.

Cryolipolysis has emerged as a new non-invasive body contouring method using controlled cooling to selectively destroy fat cells without damaging to the skin and other surrounding tissues [7]. The cooling applicator is applied to the treated area in order to extract heat at a set rate (mW/cm^2^) until a target temperature is reached (e.g., −7 to 1 °C) for a pre-determined time period [8]. After a single treatment, apoptosis of adipocytes and an increase in the collagen to adipose tissue ratio by the process of lobular panniculitis and thickening of the interlobular fibrous septae occurs over a period of several months, resulting in reduction in the thickness of the subcutaneous fat layer [9]. An animal study demonstrated 30–50 % reduction in subcutaneous fat layer thickness without causing any changes in serum lipid levels [10]. Previous clinical studies indicated the safety and efficacy of cryolipolysis for the reduction of localized subcutaneous fat on the abdomen [11] and flanks [12]. The procedure has received the US Food and Drug Administration (FDA) clearance for fat reduction of flanks, abdomen, and thighs in 2010, 2012, and 2014 respectively [13].

Recently, the new flat cup vacuum (CoolFit) applicator has been developed and commercially available in Asia. The design of this new applicator included a flat applicator cup and 38 % larger cooling plates than the ordinary applicator (CoolCore) used to treat abdomen and flanks. With the new design, the longer fat bulges, such as localized subcutaneous fat on arms and inner thighs, can be treated with cryolipolysis. The clinical efficacy of inner thighs treatment has been reported [14]. However, the cryolipolysis treatment on arms by the new flat cup vacuum has never been studied.

The objective of this study was to determine the efficacy of non-invasive cryolipolysis for body contouring with the flat cup vacuum applicator on arms and inner thighs.

## Methods

This study was conducted at the Siriraj laser center, Department of Dermatology, Faculty of Medicine Siriraj Hospital, Mahidol University, Bangkok, Thailand. Twenty Thai females aged more than 20 years with excessive localized subcutaneous fat on the arms or inner thighs were recruited to the study. All subjects had skin phototypes III to V. All subjects received a single session of cryolipolysis treatment (CoolSculpting system; Zeltiq Aesthetics, Pleasanton, CA) on their arms, inner thighs, or both, at Cooling Intensity Factor (CIF) of 41.6 for 60 min followed by 2 min of manual massage on the treated area. A total of 40 areas were treated including 10 arms and 30 inner thighs. All subjects were instructed to adhere to their regular diet, exercise program, and life style with weight fluctuations not exceeding 2 kg from the preceding month. Exclusion criteria included scarring, inflammation or infection of the area to be treated, pregnancy or lactating, subjects with history of malignancy, cold urticaria, Raynaud’s phenomenon, cryoglobulinemia, paroxysmal cold hemoglobinuria, and prior treatment of the area with another body contouring method within 1 year of the baseline visit.

The patients were objectively evaluated using standardized photographs, and measurements of body weight and circumference of arms or inner thighs at baseline, 3-month, and 6-month follow-up visits. Standardized digital photographs using consistent patient positioning, camera angling, and lighting were obtained. Circumference measurements were done using one designated tape measure and were always taken at a consistent distance from an anatomical landmark, superior distance from olecranon process for arm, and superior distance from upper pole of patella for inner thigh, for future site and measurement verification. All measurements were done by the same investigator to avoid possible inter-observer variation.

Patients’ satisfaction survey were also evaluated at 3- and 6-month follow-up by using a quartile grading scale (−1 = worst, 0 = no improvement, 1 = 1–24 % improvement, 2 = 25–49 % improvement, 3 = 50–74 % improvement, 4 = 75–100 % improvement). Side effects were recorded at the treatment session and follow-up visit.

All subjects were instructed to maintain their normal lifestyle, diet, and food consumption during the entire study. No other complimentary treatment including nutritional supplement, mechanical massages, or medication were given to any subjects. This study was approved by the Ethical Committee on Research Involving Human Subjects, Faculty of Medicine, Siriraj Hospital, Mahidol University and conformed to the guidelines of the 1975 Declaration of Helsinki. Written informed consent was obtained from all study subjects.

Descriptive statistics including mean, median, minimum, maximum, percentages of circumferential reduction, and 95 % confidence interval were used to describe demographic data and circumference measurements. The mean differences of body weight and circumferences of arms or inner thighs at baseline, 3-month, and 6-month follow-up visits were analyzed by repeated measure ANOVA. All statistical data analyses were performed using statistical software (SPSS version 16.01; SPSS Inc, Chicago, Illinois).

## Results

Of all 20 patients, 17 completed the treatment and attended 3-, and 6-month follow-up. Three subjects were withdrawn from the study, 1 subject could not complete the treatment session due to pain and numbness during treatment, 1 subject became pregnant after treatment, and the other subject could not attend all required follow-up visits. A total of 34 areas were treated including 24 inner thighs (70.59 %) and 10 arms (29.41 %). The average mean age of subjects was 30.2 years with a standard deviation of 5.8 years (range 25–50 years). All subjects had Fitzpatrick skin type IV. The average body mass index (BMI) was 21.15.

**Fig. 1:**
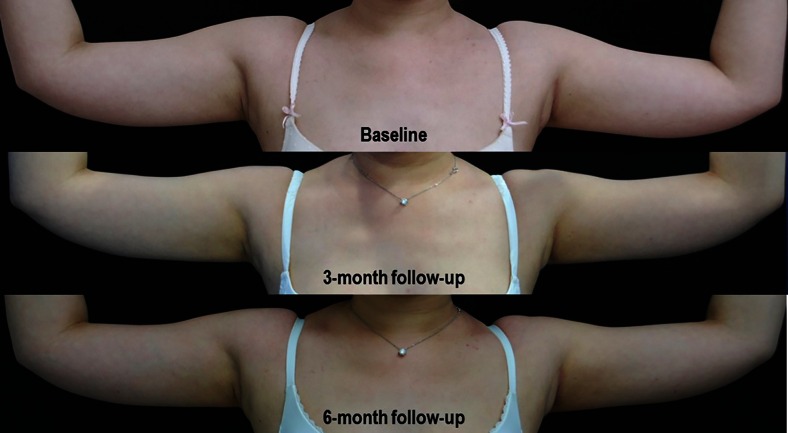
Clinical improvement of arms at baseline, 3-, and 6-month follow-up

**Fig. 2:**
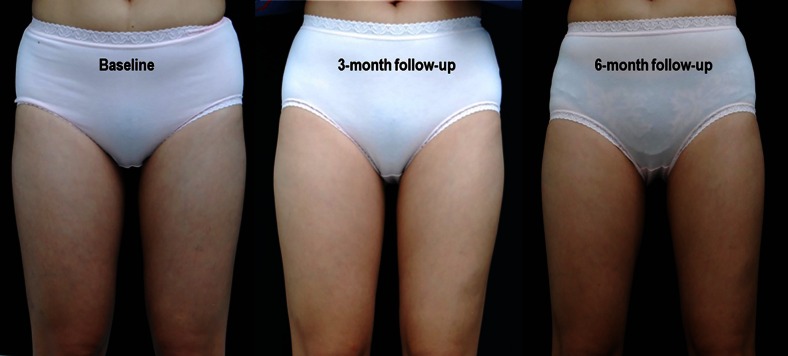
Clinical improvement of inner thighs at baseline, 3-, and 6-month follow-up

The average body weights of all subjects at baseline, 3-, and 6-month follow-up visits were 55.65, 55.03, and 55.96 kg, respectively. There was no statistically significant change in weight at 3- and 6-month follow-up visits when comparing to baseline. In contrast, the circumference of treated areas was reduced significantly at 3- and 6-month follow-up visits (Table 1). The average circumference reductions at 3- and 6-month follow-up visits were 0.41 and 0.72 cm, respectively, accounting for 0.87 and 1.52 % reduction, respectively. The clinical improvements of arms and inner thighs are shown in Figs. 1 and 2.

**Table 1:** The average weight and circumference of the treated area at baseline, 3-, and 6-month follow-up

	Baseline (mean ± SD)	3-month follow-up (mean ± SD)	6-month follow-up (mean ± SD)	*p* value
Body weight (kg)	55.65 ± 6.13	55.03 ± 6.53	55.96 ± 5.99	0.317
Circumference (cm)	47.34 ± 2.06	46.93 ± 2.10	46.62 ± 2.05	0.017*

*statistically significant difference

**Fig. 3:**
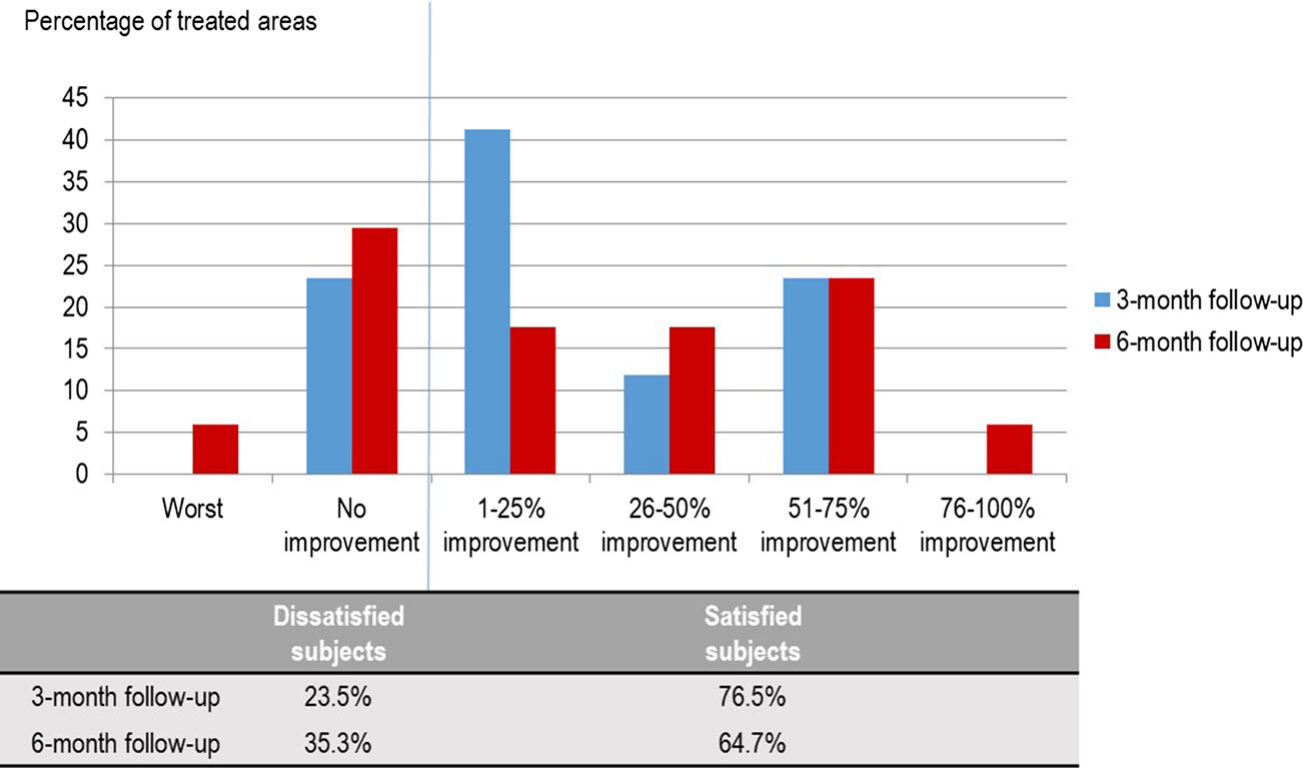
Patients satisfaction survey and percentage of patients who were dissatisfied with the results at 3 and 6 months after treatment

Patients’ satisfaction surveys showed that most of the patients were rated to have 1–25 % improvement at 3- and 6-month follow-up after treatment. There was no statistically significant difference between the satisfaction at 3-, and 6-month follow-up (*p* = 0.835). However, there was a higher percentage of subjects graded worst or no improvement (dissatisfied subjects) at 6 months when comparing to 3 months after treatment (35.3 vs. 23.5 %) as shown in Fig. 3. Therefore, additional analysis was performed by comparing the weight change at 3- and 6-month follow-up visits of dissatisfied subjects and satisfied subjects. We found that the dissatisfied subjects increased weight significantly at 6 months after treatment when comparing to satisfied subjects (*p* < 0.001). The average weight increment of dissatisfied subjects at 6 months after treatment was 1.88 kg (Table 3).

Adverse reactions of 34 treatments included pain, erythema, dysesthesia, and purpura on the treated area. The percentage and duration of side effects are shown in Table 2. Intra- and post-procedural pain was described as mild to moderate by most of the patients. Pain score was measured by visual analog scale of 0–10. The mean pain score rated by subjects was 7 ranging from 1 to 10. However, 1 subject could not bear the pain and dysesthesia during treatment on her right upper arm. The applicator was then removed before completing the session. The total treatment time was only 45 min. Post-inflammatory hypopigmentation, scarring, and paradoxical adipocyte hyperplasia were not observed in our patients.

**Table 2:** Adverse reactions from cryolipolysis found in this study

Adverse reactions	No. (%)^a^	Mean duration (days)	Median duration (days)	Range (days)
Pain	14 (41.2 %)	7	7	1–14
Erythema	24 (70.6 %)	12	7	1–21
Dysesthesia	27 (70.6 %)	13	7	2–30
Purpura	18 (52.9 %)	9	7	3–21

^a^From all 34 treatments

## Discussion

There are several cryolipolysis applicators for treating discrete fat bulges on the flanks and abdomen, but treating longer fat bulges on arms and inner thighs is still a challenge. With the new flat cup vacuum applicator, the longer fat bulges on non-curved areas, e.g., arms and inner thighs, can be easily reached and treated. The previous pilot study demonstrated the efficacy of the new applicator on the treatment of inner thighs. At 16 weeks after treatment, fat layer reduction was attained in 83 % of patients. The mean reduction in fat layer thickness was 20 % corresponding to 3.3 mm measured by ultrasound. Most of the patients (91 %) were satisfied with the treatment outcome [15].

In this present study, 76.5 and 64.7 % of patients were satisfied with the results at 3- and 6-month follow-up, respectively. This finding was correlated with the previous study [16]. However, there was a higher percentage of dissatisfied subjects at 6 months when comparing to 3 months after treatment (35.3 vs. 23.5 %) due to average weight increment in this group (Table 3). Therefore, it is mandatory to maintain normal weigh after cryolipolysis, especially dietary control and regular exercise, to prolong the clinical improvement and satisfaction.

**Table 3:** The average weight change and patients’ satisfaction at 3- and 6-month follow-up

	The average weight increment (kg)		*p* value
	Dissatisfied subjects (worse or no improvement)	Satisfied subjects (1–100 % improvement)	
3-month follow-up	−0.25 ± 0.96	−0.94 ± 1.69	0.457
6-month follow-up	+1.88 ± 0.50	−0.40 ± 1.12	<0.001*

*statistically significant difference

**plus value represents weight gain and minus value represents weight loss

To the best of our knowledge, this is the first study reporting the clinical efficacy of cryolipolysis in the treatment of localize fat bulges on arms. In contrast to other treated sides, patients receiving cryolipolysis on arms reported a higher degree of pain and dysesthesia during treatment. One subject in this study was withdrawn because she could not bear the pain and dysesthesia during treatment on her right upper arm. The applicator was then removed at 45 min after starting the treatment. However, the circumference of her right arm decreased 0.60 cm at 3 months after treatment, and this reduction was maintained up until 6 months. Therefore, the patients who want to receive cryolipolysis treatment on arms should be warned regarding the potential pain and dysesthesia sensation during treatment session. To reduce the incidence of pain and dysesthesia caused by compression of the ulnar nerve during cryolipolysis, we take care to avoid entrapping the superficial aspect of the ulnar nerve within the vacuum applicator. When placing the applicator for an arm treatment, we position the applicator towards the lateral side of the arm.

Also, care is taken to position the applicator for inner thigh treatments to avoid treatment area demarcation. As described in a previous inner thigh study, the flat cup vacuum applicator should be placed over the treatment area slightly posterior to the thigh midline, positioned vertically, and as high as comfortably tolerated to capture the inner thigh bulge when vacuum suction is initiated [14]. Patient assessments are important, and the inner thigh treatment area should be squeezed to ensure that the targeted subcutaneous fat can be pulled into the vacuum applicator cup for an aesthetically pleasing outcome.

In summary, the present study demonstrates the new cryolipolysis flat cup vacuum applicator provided beneficial effects on the circumferential reduction of arms and inner thighs. However, weight control is still important to maintain the clinical improvement and patient satisfaction. Controlled studies with a larger number of patients and longer follow-up period are warranted to fully evaluate this treatment modality.
